# Improvement of Automatic Measurement Evaluation System for Subway Structures by Adjacent Excavation

**DOI:** 10.3390/ma14247492

**Published:** 2021-12-07

**Authors:** Jung-Youl Choi, Sun-Hee Kim, Ho-Hyun Lee, Jee-Seung Chung

**Affiliations:** 1 Department of Construction Engineering, Dongyang University, No. 145 Dongyangdae-ro, Punggi-eup, Yeongju-si 36040, Korea; jychoi@dyu.ac.kr (J.-Y.C.); jschung@dyu.ac.kr (J.-S.C.); 2 Department of Architectural Engineering, Gachon University, 1342 Seongnamdaero, Sujeong-gu, Seongnam-si 13120, Korea; 3 SANGANG ENC Co., Ltd., 1519ho, 127, Beobwon-ro, Songpa-gu, Seoul 05836, Korea; sge2012@hanmail.net

**Keywords:** automated measurement methodology, field measurement, numerical analysis, tunnel displacement, track deformation

## Abstract

This study evaluated the structural stability of subway structures based on adjacent excavations by comparing automatically measured and numerically analyzed data. The reliability of the automated measurement methodology was evaluated by first applying probability statistical analysis to the measured results and then comparing these results with the numerically analyzed results. An improvement in the calculation method evaluation system, including the method of processing and analysis of the automatically measured data of subway structures through the average value of probability density, was proposed. As a result of the field measurement and numerical analysis, the measured results of tunnel displacement and track deformation exhibited some differences. However, it was determined that the construction stage and location where the maximum values of the tunnel displacement and track deformation occurred had similarities.

## 1. Introduction

With the increasing urban population and the consequent increase in demand for high-rise buildings, large-scale excavation works at greater depths have increased in areas adjacent to existing subway structures. For ground excavations in city centers, ensuring safety during the excavation process is highly important not only for earth-retaining structures but also for the adjacent subway structures that are responsible for large-scale public transportation. Although the use of measurement systems for the maintenance of subway structures, expressway tunnels, and high-speed rail tunnels has gradually increased, the level of utilization of the measurement results is low compared with the investment cost and the efforts of engineers. In addition, safety evaluation of subway structures and tracks with adjacent excavation is mostly performed with respect to geotechnical aspects, and there have been limited studies on accurate measurement and analysis methods for the measured data.

In Korea, Park and Lee [1] used laser scanning to investigate the tunnel liner behavior following the excavation of the upper layers, and Bae et al. [2] presented a performance evaluation method for the evaluation of a tunnel scanner used for tunnel crack detection.

In studies abroad, Feng et al. performed a feasibility analysis of two measurement methods (TDP&S and TDP-S) used for microseismic monitoring through numerical experimentation [3]. Manuello et al. conducted a real-time investigation of the damage in a precast concrete arch tunnel using a multichannel acoustic emission acquisition system. In addition, the active cracks were localized based on finite element analysis [4]. Miliziano et al. predicted the effect of tunneling on Roman buildings through the numerical analysis with the Mohr–Coulomb model using parameters [5]. Yang et al. proposed an automation method in which parameters were adjusted to obtain the optimal parameters of a B-spline model for the optimization of a composite tunnel structure model. An automatic and intelligent modeling method for composite tunnel structures was investigated based on the maximum likelihood estimation method. As a result, the overfitting phenomenon was analyzed using the B-spline approximation [6]. Zhou et al. [7] performed automated monitoring of the subway tunnel displacement based on the use of a robotic total station (RTS) using prisms as reflectors. In addition, an experiment on a tunnel showed the available monitoring range of a single RTS, and the accuracy of monitoring the long zone with multiple RTSs was verified. Xue et al. performed deep leaning-based automatic recognition of the water-leakage area in a shield tunnel lining to derive an automatic and accurate calculation of the water leakage area [8]. Wang et al. [9] investigated the effect of retaining technology for deep foundation pit excavation adjacent to high-speed railways based on deformation control through field measurement results and numerical analysis. As a result, the deformation of the foundation pit and retaining wall including the railway subgrade that occurred during the excavation process was confirmed. Qin et al. used a deep convolutional network with data augmentation, and deep learning-based automatic recognition was performed for automatic recognition of tunnel lining elements using ground penetrating radar images [10]. This method yielded an accuracy of 95.45% for the initial lining thickness recognition, as confirmed by field experiments. Most previous studies have investigated the presence or absence of track deformation based on automatically measured results.

To analyze the behavior of subway structures with respect to adjacent excavation constructions, the automatically measured results obtained with probability statistical analysis were compared in this study with numerically analyzed results. The evaluation technique and improvement method were presented through a reliability evaluation of the measured results.

## 2. Numerical Analysis

### 2.1. Modeling

The numerical analysis model is a structure located in Seoul, and the 3D modeling was performed based on the 2D drawing. In this study, numerical analysis was performed on subway structures and track deformation when excavation work was conducted adjacent to subway structures that are in operation.

To investigate the stability of adjacent subway structures owing to the excavation work in the earth to retain temporary supports, a model was applied which included the retaining wall, surrounding ground, and subway structures. In addition, numerical analysis was performed using MIDAS GTS NX (version 290) [11], a general-purpose finite element analysis program for geotechnical analysis. The ground elements were composed of solid elements, axisymmetric elements, and plane-strain elements. The structural elements consisted of geogrid elements, pile elements, plane-stress elements, shell elements, beams/embedded beam elements, and trusses/embedded truss elements. Other elements consisted of infinite elements, free field elements, interface elements, elastic/rigid link elements, and spring elements.

In this study, a three-dimensional (3D) solid element was applied to analyze the behavior and stress of the ground, and the analysis was performed based on a continuum with the application of elastoplastic theory. The analysis domain included subway structures and the depth of excavation was set to elevation (E.L) (-) 19.45–19.65 m (excavation depth H = 32.57–32.65 m).

For the numerical analysis modeling of the surrounding ground, the Mohr–Coulomb model, which is generally applied to soil and rock mass, was used. After the initialization state was completed, analysis was performed for each construction process of the excavation work. The initial groundwater level was E.L (-) 16.3–15.3 m. The ground boundary condition was set as the constraint condition for displacement within the model, the left and right boundary conditions with reference to the total coordinate system were set as the roller condition, and the lower boundary conditions were set as the hinged condition.

The input properties for the ground and structural components used in the analysis are presented in Table 1 and Table 2, respectively [12,13].

The construction stages considered for the numerical analysis are presented Table 3. As shown in Table 3, the field measurement was set to a removal stage in which the surcharge load rapidly decreased, and the measured results in the corresponding stage were used for comparative analysis with the results obtained from the numerical analysis.

**Figure 1:**
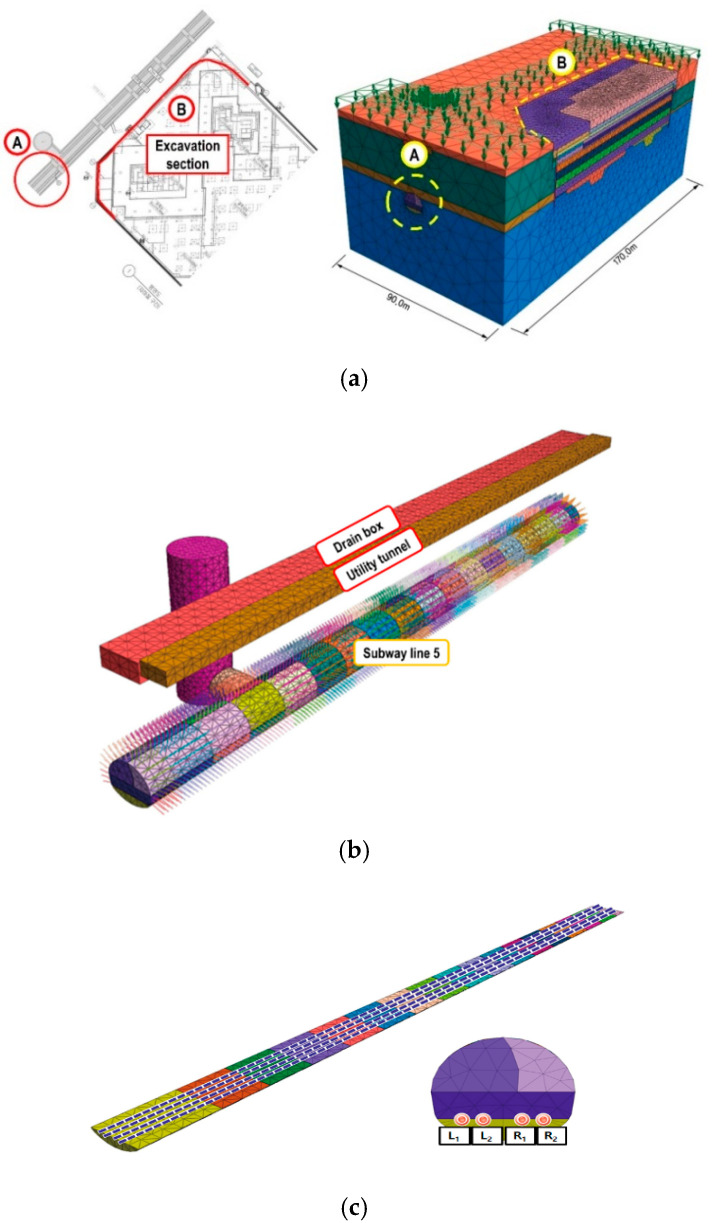
Numerical analysis modeling. (**a**) Topography and modeling of load application on the railroad; (**b**) Subway superstructure modeling; (**c**) Rail model.

To reflect the initial conditions for the existing structure to be removed and existing subway structures and the evaluation of excavation impact, the analyzed domain (170 m) was set to be wider than the length of the excavation (approximately 110 m). The details of the numerical modeling are shown in Figure 1a, and the details of the 3D continuum modeling for the superstructure of the tunnel are shown in Figure 1b. The modeling of the rail is shown in Figure 1c [14].

### 2.2. Analysis of Results

**Figure 2:**
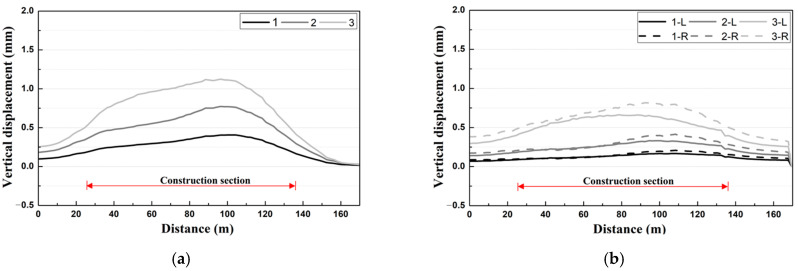
Results of tunnel displacement analysis according to the construction stage. (**a**) Vertical displacement; (**b**) Horizontal displacement.

In this study, to investigate the tunnel stability characteristics with respect to adjacent excavation, two items of vertical displacement and horizontal displacement were compared with the respective reference values. As shown in Figure 2a [14], the maximum vertical displacement for each removal stage is upward displacement up to 1.124 mm, indicating increases of approximately 90–176% compared with the initial value. In Figure 2b, the maximum horizontal displacement for each removal stage is up to 1.124 mm, which is equivalent to the rate of increase of approximately 99–301% against the initial value.

**Figure 3:**
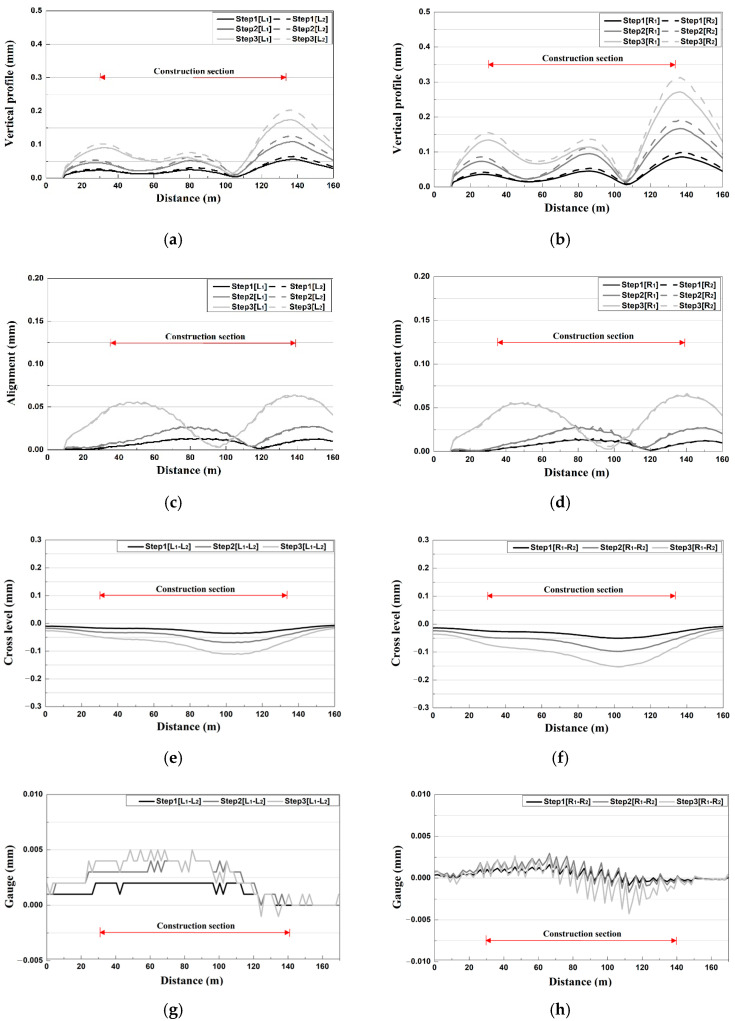
Results of track irregularity analysis at different construction stage. (**a**) Vertical profile (out-of-plane distortion) (upward track); (**b**) Vertical profile (out-of-plane distortion) (downward track); (**c**) Alignment (lateral misalignment) (upward track); (**d**) Alignment (lateral misalignment) (downward track); (**e**) Cross level (up track); (**f**) Cross level (down track); (**g**) Gauge (up track); (**h**) Gauge (down track).

As shown in Figure 1c, the tracks were divided into left and right tracks, and the left and right rails of the left track were indicated as L_1_ and L_2_, and the left and right rails of the right track were indicated as R_1_ and R_2_, respectively [14]. Various types of track irregularities of the subway track (vertical profile, alignment, cross level, and gauge) were analyzed. As a result of the vertical profile analysis, as shown in Figure 3a,b, in the initial stage, the left and right track deformation levels were similar, but in the process of removal, differences in the vertical profiles of the left and right tracks occurred. As shown in Figure 3b, the vertical profile was found to be higher in the right track close to the construction site. In addition, marked changes were observed at the ends of the construction section, and the level of the vertical profile at the center of the construction section was very low. The vertical profile, which evaluates the difference in relative displacement of the left and right rails for a rail length of 10 m, was analyzed to show large values at locations with a significant change in the generated displacement, such as the ends of the construction section and the starting point of deformation of the structure.

As a result of the alignment analysis, a clear increase in alignment was observed at the ends of the construction section for the left track, as shown in Figure 3c [14]. For each removal stage, the alignment values increased by approximately 108–392% compared with the initial value, and the value was rather low at the center of the construction section. For the right track, as shown in Figure 3d, the alignment exhibited a similar behavior to that of the left track and yielded a significant increase at the ends of the construction section.

As a result of the cross-level irregularity analysis, for the left track, the maximum value was observed at the center rather than at the ends of the construction section, as in the case of the gauge, as shown in Figure 3e. In addition, although a small displacement was generated at Stages 1 and 2, a clear increase was observed at Stage 3. In the case of Figure 3f, which is the right track, the overall trend of the cross level was similar to that of the left track, but larger values of cross level were shown for the right track, which is closer to the construction site compared with the left track.

As a result of the gauge irregularity analysis, unlike the case of the vertical profile and alignment, small changes were generated at the ends of the construction section for the left track, as shown in Figure 3g. For each removal stage, the gauge irregularity was decreased by approximately 25% compared with the initial value, and the maximum value was observed at the center of the construction section. As shown in Figure 3h, the gauge was relatively large on the right track, close to the construction site.

## 3. Field Measurements

### 3.1. Automated Field Measurement Method

In order to confirm the structural safety of the adjacent subway structure during new construction in Yeouido, Seoul, a sensor was attached to the position of the maximum displacement obtained through the numerical analysis to confirm the deformation of the tunnel.

Tunnel deformation of subway structures occurs due to excavation of structures adjacent to subway structures. In order to check the abnormal displacement at an early stage by measuring the displacement state of the structure, a tunnel convergence meter was installed in the subway tunnel structure. In addition, the rail bed settlement monitoring sensor is a high-resolution sensor that measures the amount of displacement on the track, such as a railway subgrade, and measures the stability of adjacent structures. The exact amount of settlement is measured by measuring the displacement of the track.

For field measurements, the displacements generated by the adjacent excavation were monitored by automated measurements at 60 min intervals by installing tunnel convergence meters (tape extensometer, TL) and rail bed settlement monitoring sensors (RM) in subway structures and tracks, respectively, and the measured data were compared with the numerically analyzed results.

**Figure 4:**
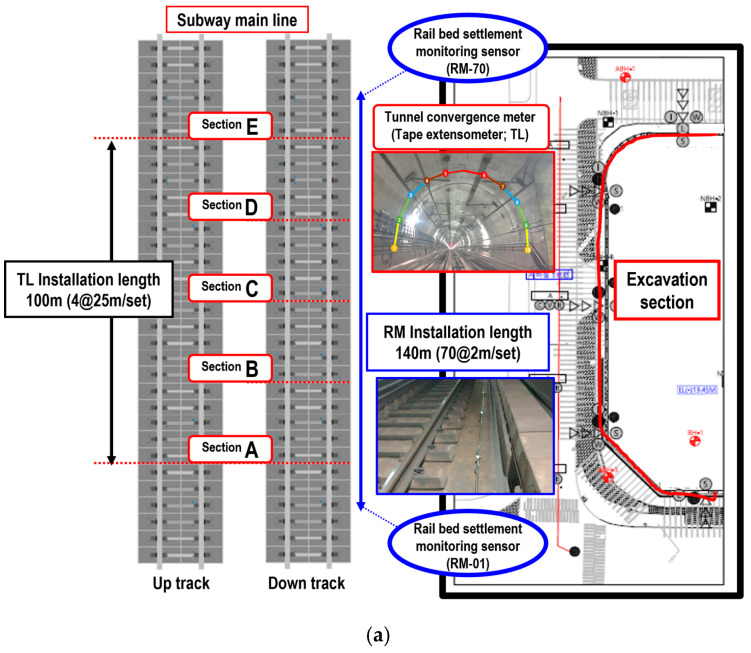

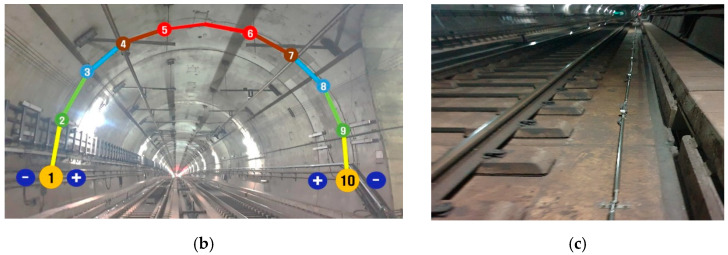
Sensor installation section and front view. (**a**) Schematic of automated measurement sensor installation plan; (**b**) Front view of tunnel convergence meter (TL) sensor installation; (**c**) Front view of STL sensor installation.

To install the tunnel convergence meters for tunnel displacement measurement, 10 sensors for each section were installed in five sections at 25 m intervals in a 100 m section, as shown in Figure 4a. As shown in Figure 4b, the tunnel convergence meters TL-1 and TL-10 were installed in the left and right haunches of the tunnel to measure the vertical and lateral displacements, TL-2 to 4 and TL-7 to 9 were installed on the tunnel lining wall and measured vector displacements, and TL-5-6 were installed at the arch of the tunnel and measured vertical displacements. RMs were installed in the subway main section to measure the track displacement, as shown in Figure 4a [14]. A total of 70 sets of vertical displacement sensors were installed at intervals of 2 m in a section with a total length of 140 m. Table 4 lists the specifications of the measurement sensors used in this study.

### 3.2. Field Measurement Results

**Figure 5:**
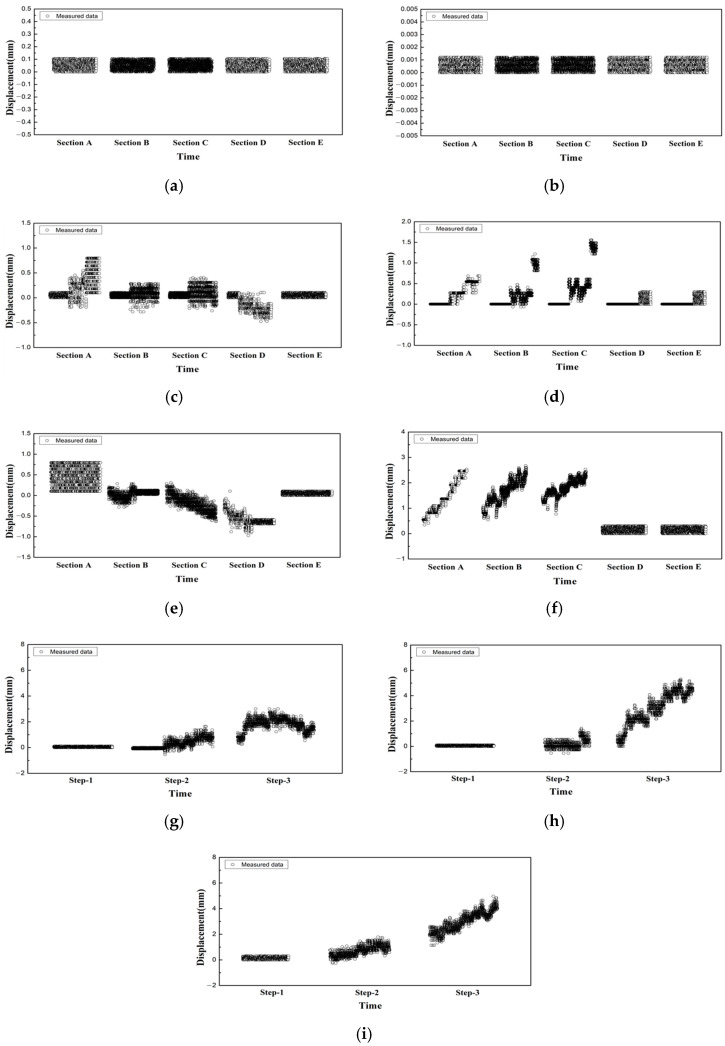
Example of tunnel and track displacement measurements. (**a**) Removal stage 1 (TL-6, vertical displacement); (**b**) Removal stage 1 (TL-10, lateral displacement); (**c**) Removal stage 2 (TL-6, vertical displacement); (**d**) Removal stage 2 (TL-10, lateral displacement); (**e**) Removal stage 3 (TL-6, vertical displacement); (**f**) Removal stage 3 (TL-10, lateral displacement); (**g**) Track displacement by removal stage (RM-11); (**h**) Track displacement by removal stage (RM-36); (**i**) Track displacement by removal stage (RM-59).

The results of the tunnel displacement measurement at different sections are shown in Figure 5a–f. In the removal stage 1, small and constant changes were measured over time in the vertical and lateral displacements of all sections, as shown in Figure 5a,b. As shown in Figure 5c–f, the displacement clearly increased after the removal stage 2 [14]. The vertical displacement (TL-6) showed large variation over time in Sections B to D, and the variation in the displacement at removal stages 2 and 3 was approximately 0.5 mm. Conversely, lateral displacement (TL-10) measurements showed large variations over time in Sections A to C, and the variation in the displacement at removal stages 2 and 3 was approximately 1 mm.

Examples of the track displacement measurement results are shown in Figure 5g–i. For removal stage 1, a similar behavior to the tunnel displacement measurements can be observed. After removal stage 2, there was a clear increasing displacement trend, and in stage 3, the most significant increase in displacement was observed. As shown in Figure 5h, RM-36, a sensor installed adjacent to the excavation section, showed the most significant increase in displacement, and its value was larger than the measurement result of the tunnel convergence meter (TL-10).

### 3.3. Analysis of Tunnel Convergence Measurement Results

In this study, as shown in Figure 5 [14], the displacement responses over time measured by automated measurements were analyzed by the removal stage. By applying the time-series displacement response signal processing method for each construction stage, the time or construction stage of the occurrence of the most significant deformation of subway structures and tracks during the construction process can be derived.

**Figure 6:**
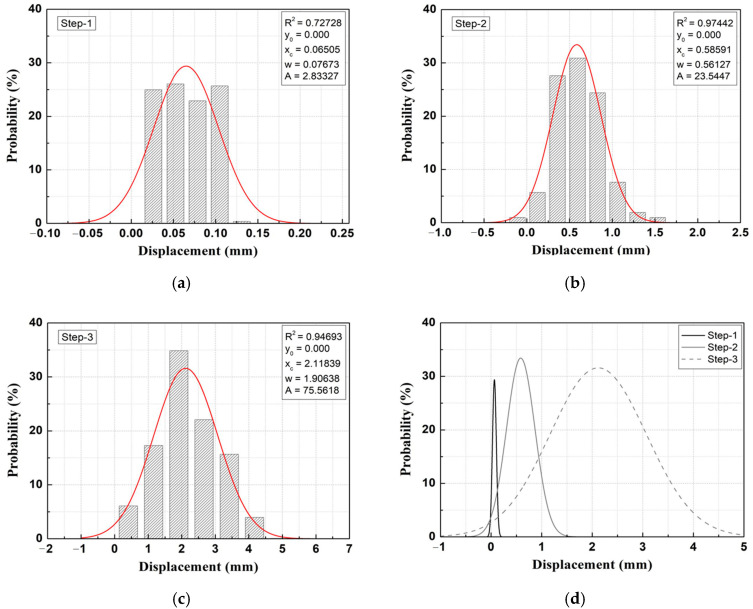
Gaussian probability density function (PDF) analysis of measured data acquired from the rail bed settlement monitoring sensor (RM) (Example). (**a**) Removal stage 1; (**b**) Removal stage 2; (**c**) Removal stage 3; (**d**) Removal stages 1, 2, and 3.

Figure 6 shows an example of a Gaussian probability density function (PDF) analysis of the automated measurement data as a function of the removal stage.

As a result of Gaussian PDF analysis using time-series displacement measurements (automated measurement data) for each construction stage, as shown in Figure 6 [14], the measurement data for each construction stage appeared in the form of a normal distribution with a small standard deviation, thus indicating that the automated measurement data have secured a sufficient level of reliability. In addition, as shown in Figure 6d, the mean value (x_c_) of the Gaussian PDF for each removal stage increases, and the standard deviation (range) of the displacement generated in removal stage 3 is large, thus indicating that the construction stage, which has a direct impact on the behavior of the structure, is removal stage 3.

**Figure 7:**
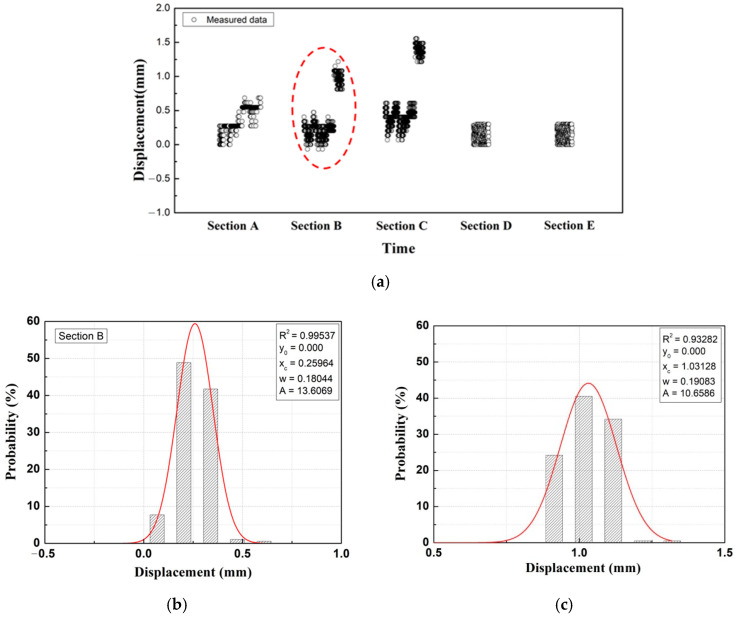
Gaussian PDF analysis (Example). (**a**) Measured data; (**b**) Section B–1; (**c**) Section B–2.

As shown in Sections B and C of Figure 7a, the measured results at the same point are divided in two groups. This indicates that even at the same location and construction stage, there is a time point in the construction process when the structure deforms, and deformation of adjacent areas occur simultaneously in the construction process. That is, it determined the range of impact on the deformation of the structure at a specific time point of construction and the time point in the construction process when the deformation of the structure occurred, which can be utilized in the determination of structural behavior during the construction process.

Based on the measured results that were divided into two groups at certain locations, it can be concluded that evaluation by simple arithmetic average or by using maximum values for measured data before the specific construction time point and the data obtained after the time point with marked displacement behavior would lead to an underestimation of the actual behavior of the structure. In addition, if time-series analysis of the displacement responses of the automated measurement data presented in this study was performed, the sudden deformation of the structure could be identified, which is expected to derive a clearer evaluation of the construction stage or construction time point that has impacted the damage of the structure.

**Figure 8:**
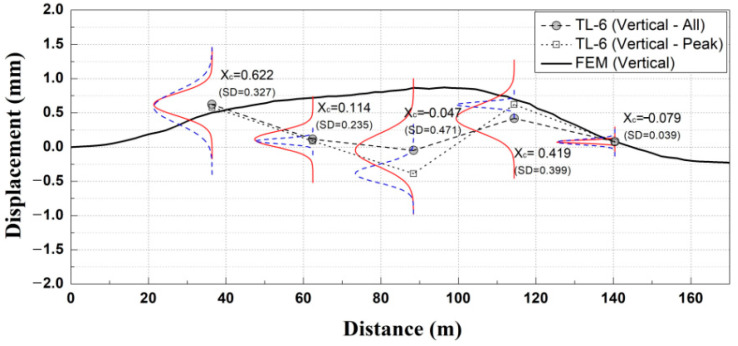
Analysis of appropriateness of the probability statistical analysis evaluation method of the measured data.

In this study, Gaussian PDF analysis was performed using the entire automated measurement data for each removal stage with the exception of the case at which the results were clearly divided into two groups, as shown in Figure 7. The numerically analyzed results obtained through the finite element method (FEM) were evaluated using the maximum value for each removal stage. Therefore, it is possible that the automatically measured results in this study, analyzed according to the time-series displacement response, may differ from the numerically analyzed results. The results of the Gaussian probability density analysis method (based on the use of all the automatically measured data for each removal stage) were compared with the analyzed results with data extracted from the main construction time points, and the occurrence of peak values were compared. The Gaussian PDF analysis results (which used all the data for each section) were compared with the Gaussian PDF analysis results (which used the peak value data for each section) at a specific construction time point (measuring period: 3 days, measured data: 72 each), as shown in Figure 8 [14].

As a result of the Gaussian PDF analysis for the peak values, as shown in Figure 8, the numerically analyzed results were shown to be within the range of the standard deviation of the Gaussian PDF, which used all the data for each section. In addition, the standard deviations of the mean values of the numerically analyzed results and the measured data were small. Thus, the probability statistical analysis evaluation method, which used all the data for each section, was analyzed to be appropriate.

**Figure 9:**
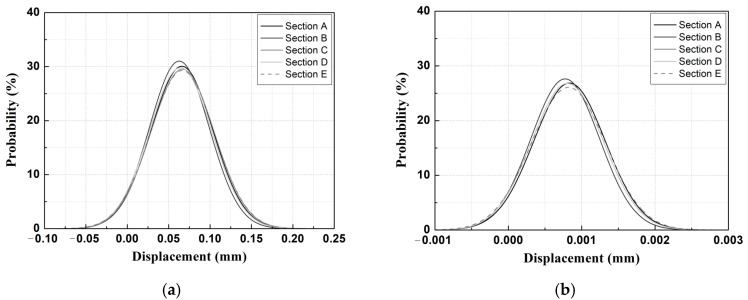

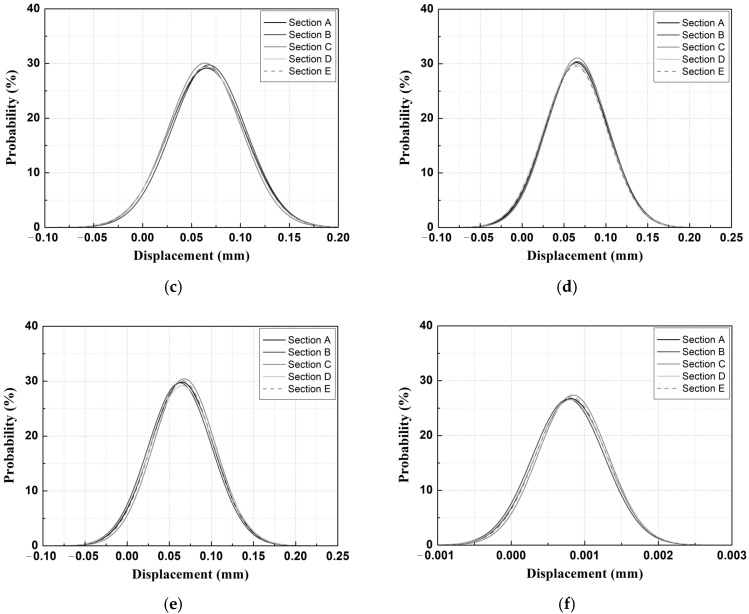
Gaussian PDF analysis results for the measured data for different TLs at removal stage 1. (**a**) TL-1 (vertical); (**b**) TL-1 (lateral); (**c**) TL-5 (vertical); (**d**) TL-6 (vertical); (**e**) TL-10 (vertical); (**f**) TL-10 (lateral).

**Figure 10:**
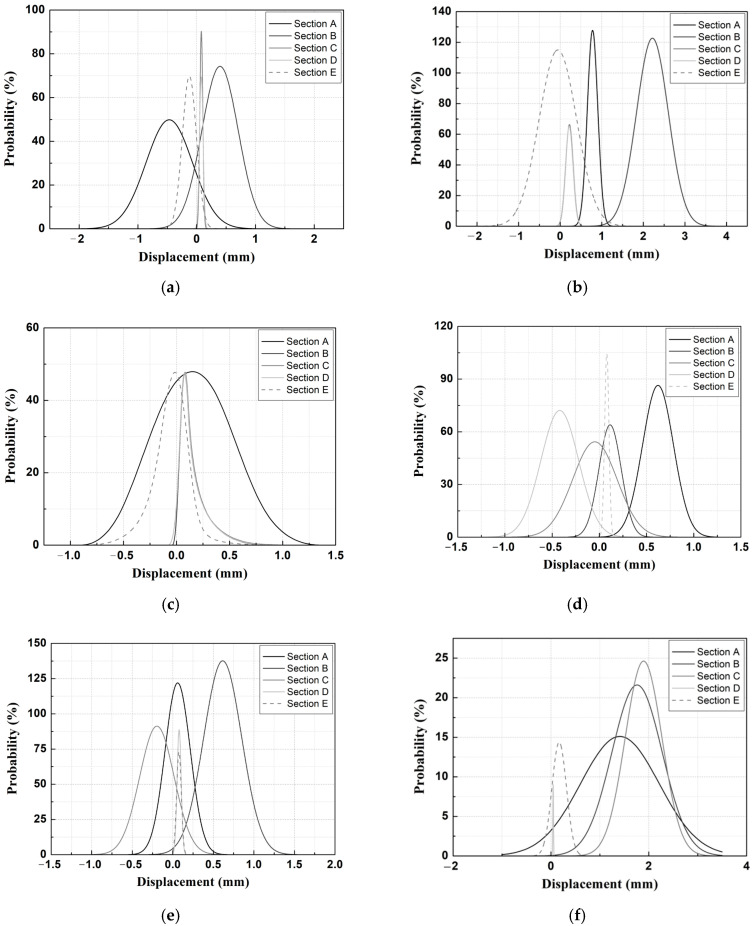
Gaussian PDF analysis results for the measured data for different TLs at removal stage 3. (**a**) TL-1 (vertical); (**b**) TL-1 (lateral); (**c**) TL-5 (vertical); (**d**) TL-6 (vertical); (**e**) TL-10 (vertical); (**f**) TL-10 (lateral).

Figure 9 and Figure 10 show the Gaussian PDF analysis results for the data measured by the TLs for different removal stages at each measurement location [14].

As a result of the Gaussian PDF analysis for each removal stage, as shown in Figure 9, the differences in the mean values (x_c_) and standard deviations of the Gaussian PDF for each sensor location and section were small in removal stage 1. Most of the Gaussian PDF analysis for the displacement measured by the TLs in removal stage 1 showed a similar distribution in the range of −0.10 to 0.20 mm. TL-1 (vertical), TL-1 (lateral), and TL-10 (lateral) were located at each end of the tunnel, and the displacement values were in the range of −0.001 to 0.0025 mm, thus showing large differences from most of the other displacement values.

The overall trends showed that the differences in the mean values (x_c_) and standard deviations of the Gaussian PDF as a function of the specific section for each TL were small, but the differences in the standard deviations of Section A of TL-8 were approximately 4%. Therefore, in the removal stage 1 of the adjacent excavation construction stages, the analyzed results showed that the impact of the subway structure sensor location and behavior by section were insignificant.

For removal stage 3, as shown in Figure 10 [14], the analyzed results showed that the differences which occurred in the mean value (x_c_) and the standard deviation of the Gaussian PDF depended on the locations of the TLs and the sections. When examining the locations of the TL installations, larger differences in the mean values (x_c_) and standard deviations of the Gaussian PDF occurred in the automatically measured data of TL-1 (lateral displacement) and TL-10 (lateral displacement) compared to TL-1 (vertical displacement) and TL-10 (vertical displacement). Therefore, in the case of the excavation section of this study in which the location of the adjacent excavation was on one side of the subway structures, the behavioral characteristics of the structure in cases in which adjacent excavations were conducted had a more direct impact on the lateral behavior than the behavior in the vertical direction.

In addition, the differences in the mean values (x_c_) and standard deviations of the Gaussian PDF for each section of TL-5 and TL-6 were large. The Gaussian PDF becomes narrower when the standard deviation is small, and it becomes wider as the standard deviation increases. Therefore, in removal stage 3 (compared with the other construction stages of adjacent excavation), the result was affected by the subway structure sensor location and behavior exhibited by each section.

## 4. Comparative Analysis of Field Measurements and Numerically Analyzed Results

### 4.1. Tunnel Convergence Analysis Results

In this study, the mean PDF values (x_c_) of the Gaussian using the TL data measured for each removal stage and numerical analysis results were comparatively analyzed.

The lateral displacements measured by TL-1 and TL-10 and the vertical displacements measured by TL-5 and TL-6 for each section were selected for comparison with the arch vertical displacement and lateral displacement at the bottom from the numerically analyzed results.

**Figure 11:**
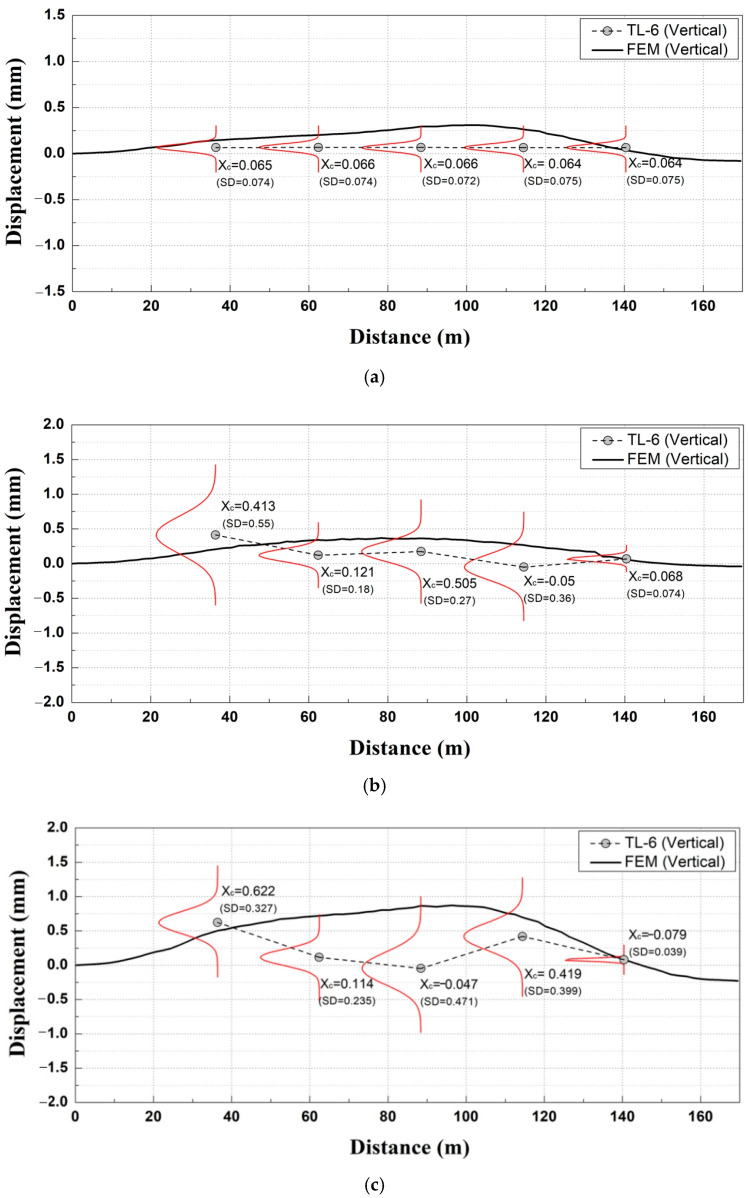
Comparative analysis of measurement data of tunnel convergence meter and numerically analyzed (vertical displacement) results. (**a**) Removal stage 1; (**b**) Removal stage 2; (**c**) Removal stage 3.

As shown in Figure 11a, the comparative analysis between the TL data and numerical analysis results (vertical displacement) at removal stage 1 yielded similar tunnel convergence values at different sections.

As shown in Figure 11b, the comparative analysis between the TL data and numerical analysis results (vertical displacement) at removal stage 2 exhibited small variations (increment/decrement) at different sections in the field measurement values of tunnel convergence.

In Section C, the tunnel convergence value yielded the largest difference from the numerically analyzed results among different sections; in terms of the overall trend, the field-measured tunnel convergence was approximately 60% smaller than the numerically analyzed results, thus indicating that a relatively small difference occurred compared to the outcomes of removal stage 1.

As shown in Figure 11c, the comparative analysis between the TL data and numerically analyzed results (vertical displacement) at removal stage 3 exhibited clear variational differences (increment/decrement) for the field-measured tunnel convergence. The measured tunnel convergence was found to be approximately 89% smaller than the numerically analyzed results. Additionally, in the case of removal stage 2, the field-measured displacement for each section was smaller than the numerically analyzed results with the exception of the tunnel convergence in Section D. Since the difference between the measured tunnel convergence and numerically analyzed results (vertical displacement) exhibited a clear trend of variation at different sections at removal stage 3, it was found that the actual impact on the deformation of subway structures was more significant at removal stage 3 compared with previous stages.

**Figure 12:**
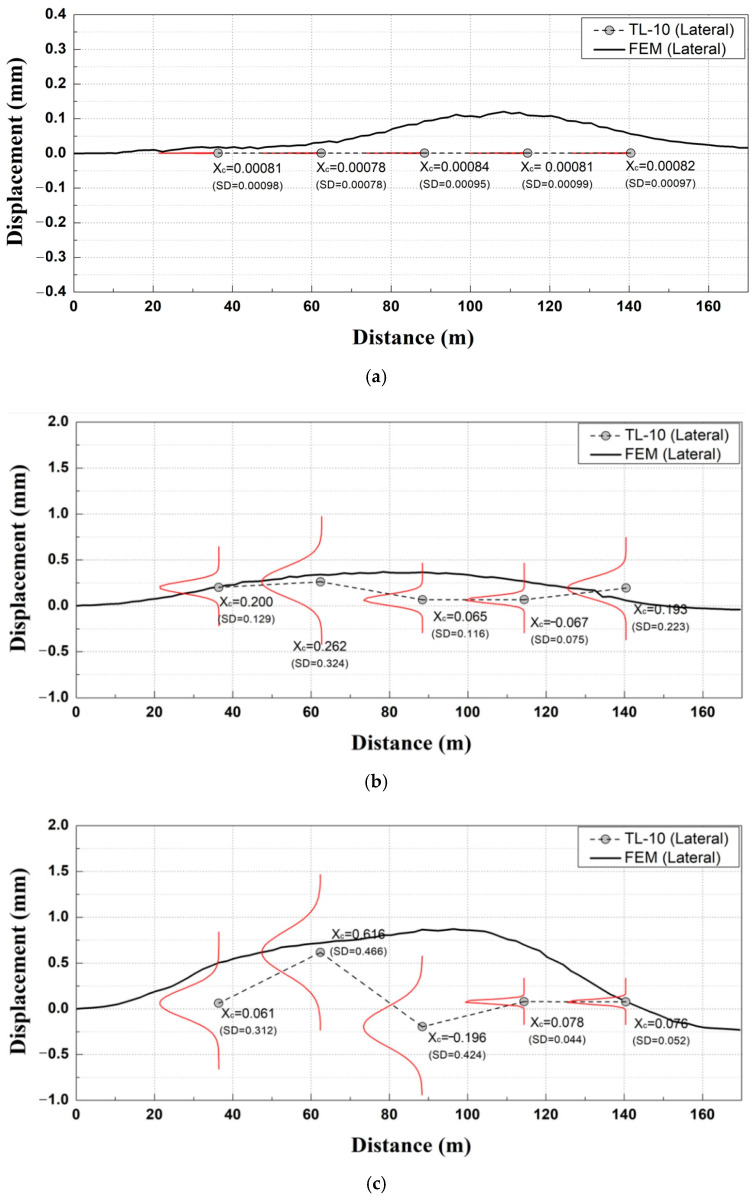
Comparative analysis of measurement data of tunnel convergence meter and numerically analyzed (lateral displacement) results. (**a)** Removal stage 1; (**b**) Removal stage 2; (**c**) Removal stage 3.

As shown in Figure 12a, the comparative analysis between the TL data and numerically analyzed results (lateral displacement) at removal stage 1 yielded similar values of tunnel convergence for each section.

As shown in Figure 12b, the comparative analysis between the TL measurement data and numerically analyzed results (lateral displacement) at removal stage 2 showed that the tunnel convergence value yielded the largest difference from the numerically analyzed results in Section C compared to the other sections. In terms of the overall trend, the field-measured tunnel convergence was approximately 55% smaller than the numerically analyzed results, thus indicating that a relatively small difference occurred compared to removal stage 1.

As shown in Figure 12c [14], the comparative analysis between the TL data and numerically analyzed results (lateral displacement) at removal stage 3 showed that the field-measured tunnel convergence was approximately 62% smaller than the numerically analyzed results. At dismantle stage 2 of the adjacent excavation process, the field-measured displacement by section was smaller than the numerical analysis result, except for the tunnel convergence meter in Section D. The numerical analysis is performed during design. There is a difference between numerical analysis and the actual behavior because there is much uncertainty about the soil conditions. Therefore, it is possible to confirm that it is safe to accurately determine the behavior of the structure by checking it through automated measurement rather that the numerical analysis.

### 4.2. Track Deformation (Rail Bed Settlement) Analysis Results

In this study, the mean PDF value (x_c_) of the Gaussian of the RM data measured at different removal stages and the numerical analysis results were comparatively analyzed. In the case of RMs, a total of 70 sensors were installed, and measurements were conducted over a total extension of 140 m.

**Figure 13:**
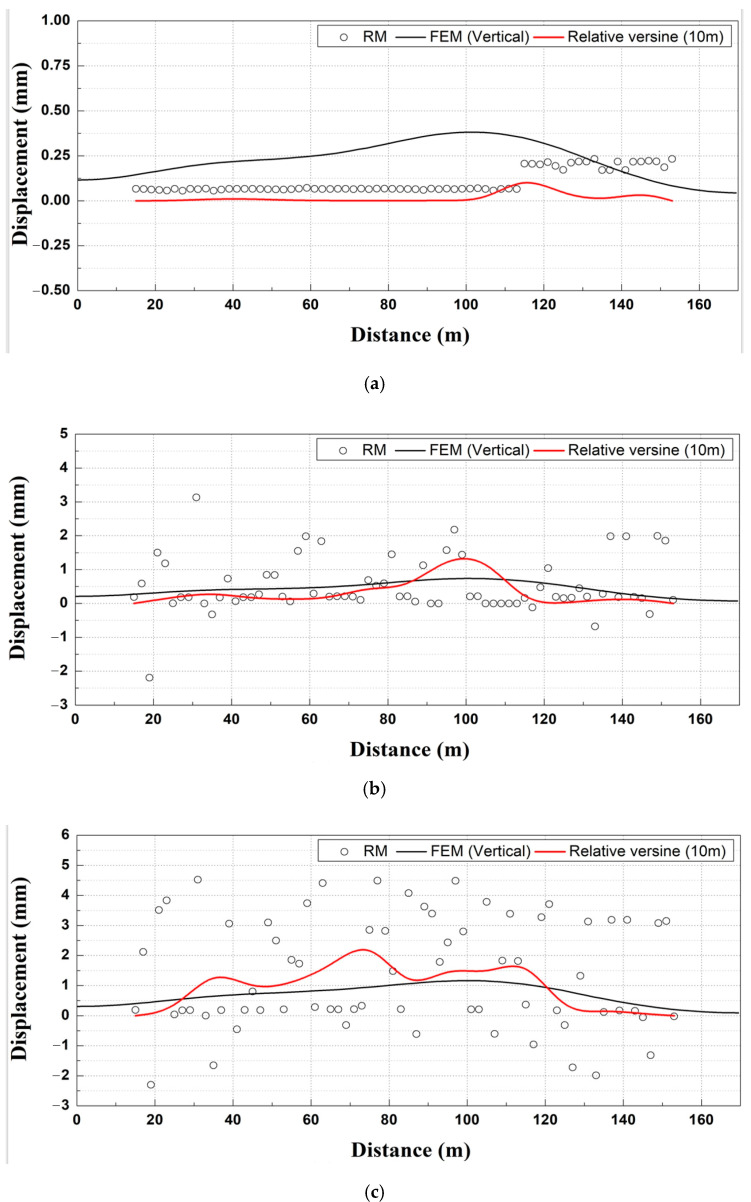
Track displacement (rail bed settlement monitoring sensor) analysis outcomes. (**a**) Removal stage 1; (**b**) Removal stage 2; (**c**) Removal stage 3.

As shown in Figure 13a, the results of the relative displacement analysis (based on an extension length of 10 m) of the mean value (x_c_) of the Gaussian PDF yielded a smaller displacement value compared to the numerically analyzed results.

For track displacement (RM) at removal stage 2, as shown in Figure 13b and the track displacement at removal stage 3, as shown in Figure 13c [14], the relative displacement analysis (based on 10 m) result of the Gaussian mean PDF value (x_c_) was similar to the numerical analysis result. Therefore, because of the track displacement analysis (RM) at different removal stages, the relative displacement analysis result (based on 10 m) of the Gaussian mean PDF value (x_c_) was similar to the numerical analysis result, thus indicating that the result was appropriate.

## 5. Conclusions

Due to the recent increase in urban development, this study analyzed the effect of excavation work adjacent to existing subway structures using numerical analysis and field measurements. To improve the reliability of the measured results of the automated measurement system for structures introduced in field measurements, an analysis and evaluation system for automated measurement data was proposed. The results were compared with the results of the numerical analysis, and the key findings are summarized as follows:

(1) The numerical analysis of the deformation of subway structures showed that the increase in the vertical displacement of the tunnel generated in the removal stage of the existing structure was similar to that of the excavation stage, and the increased value returned to the initial value in the backfill stage. Therefore, the analysis showed that in the removal stage of the existing structure, displacement management was as important as the displacement management in the excavation stage. In addition, it was found that the variation in displacement generated at the ends of the excavation section was the largest, and the maximum value occurred at the center of the excavation section.

(2) The result of the numerical analysis of track deformation indicated that in terms of the subway track deformation, even in the case of tracks in the same tunnel structure, the magnitude of the track irregularity items was directly affected by the proximity to the construction site. The alignment and vertical profiles exhibited a sharp increase at the ends of the construction section, but they were very low at the center of the section

(3) The time-series analysis of displacement responses for the automatically measured data in this study confirmed that there was a time point during the construction process when the deformation of the structure occurred, even in the same construction stage at the same location, and deformation of adjacent areas could potentially occur simultaneously in the corresponding construction process

(4) In the case of the excavation section of this study, the adjacent excavation site was located on one side of the existing structure; therefore, the measured data and analyzed results both showed that the deformation characteristics of the structure owing to the adjacent excavation were mainly observed in the lateral behavior. The analyzed results of this study showed that when the adjacent excavation site was located directly above the existing subway structures, the main direction of deformation of the structure was along the vertical direction, and when it was located on one side, the main deformation was along the lateral direction.

## Figures and Tables

**Table 1:** Input properties of soil analysis.

Category	Landfill	Sedimentary Sand	Sedimentary Gravel	Weathered Soil	Weathered Rock	Soft Rock
Material properties	Isoparametric three-dimensional (3D)	Isoparametric 3D	Isoparametric 3D	Isoparametric 3D	Isoparametric 3D	Isoparametric 3D
Model	Mohr–Coulomb					
Unit weight (kN/m^3^)	18.6	17.9	19.1	19.2	21.1	24.1
Cohesion (kPa)	0	0	0	28	30	50
Friction angle (°)	0	27	32	30	32	37
Modulus of deformation (kN/m^2^)	10,000	10,000	70,000	150,000	250,000	1,000,000
Poisson’s ratio	0.4	0.38	0.35	0.32	0.3	0.25

**Table 2:** Input properties of structural components.

Category	Slurry Wall	Concrete	Slab	Anchor, R/B	Cast in Place (C.I.P), Side Pile	Buttress	Lining
Material properties	Isoparametric 3D	Isoparametric two-dimensional (2D)	Isoparametric 2D	Isoparametric one-dimensional (1D)	Isoparametric 2D	Isoparametric 2D	Isoparametric 2D
Model	Elastic	Elastic	Elastic	Elastic	Elastic	Elastic	Elastic
Unit weight (kN/m^3^)	24	23.54	23.54	76.98	24.52	23.54	24
Modulus of deformation ((kN/m^2^)	2.78 × 10^7^	2.38 × 10^7^	2.38 × 10^7^	2.05 × 10^8^	1.79 × 10^7^	2.38 × 10^7^	2.58 × 10^7^
Poisson’s ratio	0.167	0.167	0.167	0.3	0.167	0.167	0.3

**Table 3:** Overview of construction stages.

Stages	Construction Stage	Load
Removal stage 1	Removal of the 1st floor of the existing structure + Installation of row 1 and row 2 anchors	Decrease in surcharge load
	Removal of the (underground) 1st floor of the existing structure + Row 3 anchor installation	
Removal stage 2	Removal of the (underground) 2nd floor of the existing structure + Row 4 anchor installation	
Removal stage 3	Removal of the (underground) 3rd floor of the existing structure	

**Table 4:** Sensor specifications.

Category	Tunnel Convergence Meter (Tape Extensometer)		Rail Bed Settlement Monitoring Sensor	
	Angle	Length	Angle	Length
Measurement range	±60	0–50 mm	±15°	0–50 mm
Applied voltage/total resistance	5–15 V direct current	10 kΩ	5–15 V DC	5 kΩ ± 20%
Linearity	0.05°	±1%	±0.1°	±1%
Sensitivity	0.001°	0.01% maximum	0.001°	0.001 mm
Operating temperature of sensors	−20–+80 ℃	−20–+80 ℃	−30–+65 ℃	−20–+80 ℃

## Data Availability

Data sharing is no applicable to this article.
